# Tuberculin Test Demanded

**Published:** 1900-04

**Authors:** 


### TUBERCULIN TEST DEMANDED.

TOPEKA, KAS., March 1, 1900.

*To His Excellency W. E. Stanley, Governor of Kansas:*

For the better protection of the domestic animals of the State of Kansas, and to prevent the spread of contagious and infectious diseases among the same, we herewith report to your Excellency the fact that tuberculosis, a dangerous and contagious disease, prevails to a greater or less extent among the cattle constituting the dairy and breeding herds of the States hereinafter named, and that cattle affected with this disease are being brought into the State of Kansas, and are liable to communicate said disease to other cattle with which they are brought in contact. Therefore, we respectfully request your Excellency to issue a proclamation prohibiting the importation of cattle from the States hereinafter named, except in accordance with the rules and regulations adopted by this Board and herewith submitted to you for your approval.

M. C. Campbell, Taylor Riddle, F. H. Chamberlain, members Live-stock Sanitary Committee.

PROCLAMATION BY THE GOVERNOR.

WHEREAS, The Live-stock Sanitary Commission of the State of Kansas on March 1, 1900, adopted the following rules and regulations:

WHEREAS, The Live-stock Sanitary Commission of the State of Kansas have ascertained that a great many of the breeding and dairy cattle in the States of Maine, New Hampshire, Vermont, Massachusetts, Rhode Island, Connecticut, New York, New Jersey, Delaware, Pennsylvania, Ohio, Kentucky, Tennessee, Indiana, Michigan, Illinois, Wisconsin, Minnesota, Iowa, and Nebraska are infected with a contagious and infectious disease in cattle known as tuberculosis, and that some of said cattle are being shipped into the State of Kansas for breeding and dairy purposes, it is, therefore, ordered:

*First.* That from and after this date it shall be unlawful for any cattle to be shipped, driven, or transported from the above-named States into the State of Kansas for breeding or dairy purposes; provided, however, that shipments may be made from such States into the State of Kansas for breeding and dairy purposes after said cattle have been examined and found free from tuberculosis, and a permit and bill of health given by a veterinarian of the United States Bureau of Animal Industry or a veterinarian acting under the order and direction of the live-stock sanitary board of any of the above-named States, and the certificate so given by such veterinarians shall be given in duplicate, the original of which shall be forwarded to the Secretary of the Live-stock Sanitary Commission, Topeka, Kansas, and the duplicate given to the railroad company, to be attached to the bill of lading for said cattle. And no railroad company shall accept any such cattle, nor bring nor ship any such cattle into the State of Kansas from any of the above-named States, for breeding or dairy purposes, without the certificate and bill of health herein provided for; and no railroad company shall accept from its connecting lines any cattle shipped in violation of this provision.

*Second.* Provided, however, that native cattle of the State of Nebraska may be moved into the State of Kansas upon the owner or person in charge thereof making affidavit, stating, in substance, that said cattle are natives of said State, and that said cattle have not been in any of the States above named for a year immediately preceding the making of said affidavit. Said affidavit shall be made before some officer authorized to administer oaths, and the above affidavit so made shall be given in duplicate, the original of which shall be forwarded to the Secretary of the Live-stock Sanitary Commission, Topeka, Kansas, and the duplicate shall be given to the owner or person in charge of said cattle, to be attached to the bill of lading for said cattle, or carried by the owner or person in charge when driven in; and no railroad company shall accept any such cattle for shipment, nor bring nor ship any such cattle into the State of Kansas for breeding or dairy purposes from the State of Nebraska, nor accept from its connecting lines any cattle shipped in violation of this provision.

*Third.* Cattle brought into Kansas from any of the above-named States for the purpose of exhibition at county, district, or State fairs shall not be subject to the above regulations; provided, however, that in the event sales shall be made from such exhibition, and the cattle destined to points in Kansas, the animal sold shall be submitted to the tuberculin test before the sale is consummated or the cattle moved or shipped to their destination. In case the test should show any such animals to be affected with tuberculosis, a permit for shipment to any point in this State shall not be granted.

*Fourth.* All railroad, live-stock, transportation, and stockyard companies, and their employés, and all other persons, are hereby forbidden to transport, drive, or in any way handle cattle in Kansas, except in compliance with the foregoing rules, under the pains and penalties of the following statute:

Extract of Chapter II., Session Laws of 1884. "Section 21. Any person who shall violate, disregard, or evade, or attempt to violate, disregard, or evade, any of the . . . rules and regulations, orders, or directions of the Live-stock Sanitary Commission establishing and governing quarantine shall be deemed guilty of a misdemeanor, and upon conviction thereof shall be fined in any sum not less than $100 nor more than $5000."

Sheriffs, constables, and police officers in Kansas are hereby directed to enforce these regulations, and to report any violations of same to this Commission.

In testimony whereof, I hereby set my hand, and cause to be affixed the great seal of the State of Kansas.

Done at Topeka, Kansas, this fifth day of March, 1900.

[SEAL.] W. E. STANLEY,
Governor of Kansas.

By the Governor:
GEO. A. CLARK,
Secretary of State.

